# Drivers of food acquisition practices among adolescents in suburban food environments of Lao People’s Democratic Republic

**DOI:** 10.1080/16549716.2025.2451475

**Published:** 2025-02-03

**Authors:** Thidatheb Kounnavong, Miho Sato, Christopher Turner, Elaine Ferguson, Hongkham Xayavong, Manithong Vonglokham, Sharon E Cox, Junko Okumura, Kazuhiko Moji

**Affiliations:** a School of Tropical Medicine and Global Health, Nagasaki University, Nagasaki, Japan; b Food and Markets Department, Natural Resources Institute, University of Greenwich, London, UK; c Department of Population Health, London School of Hygiene and Tropical Medicine, London, UK; d Department of Coordination and Research Information Management, Lao Tropical and Public Health Institute, Vientiane, Lao PDR; e Department of Eco-epidemiology, Institute of Tropical Medicine, Nagasaki University, Nagasaki, Japan; f Department of Health Policy and Health System Research, Lao Tropical and Public Health Institute, Vientiane, Lao PDR

**Keywords:** Adolescents, ultra-processed foods, fruit and vegetables, healthy eating, food environments

## Abstract

**Background:**

Dietary shifts among adolescents in low- and middle-income countries are exacerbating the double burden of malnutrition. Understanding the drivers of adolescent food acquisition and consumption practices and their lived experiences of the food environment is crucial for the effective development of targeted interventions and policies.

**Objective:**

To explore drivers of food acquisition and consumption practices among adolescents from two suburban schools in the food environments of Phonhong District, Lao People’s Democratic Republic.

**Methods:**

We implemented a Qualitative-Geographical Information System methodology, featuring participatory photography, follow-up photo-elicitation interviews and focus group discussions with 30 adolescents from April to July 2022. Thematic analysis triangulated key themes from photos, maps, and transcripts.

**Results:**

Drivers of food acquisition and consumption included interactions across external, interpersonal, and intrapersonal domains. The six key themes were food availability and accessibility, product properties and convenience, peers and social media, caregivers and household practices, affordability, desirability, and autonomy, and perceptions, beliefs, and social norms. Consumption of ultra-processed foods was driven by the availability and accessibility of these affordable products in schools. By contrast, consumption of fruits and vegetables was driven by parental food practices at home.

**Conclusion:**

A comprehensive multi-scalar approach is required to improve adolescent diets and nutrition in the suburban food environment of Lao PDR. This includes restricting the sale of ultra-processed foods in schools, promoting home gardening, increasing caregivers’ awareness and engagement with adolescents about the benefits of healthy food choices, and leveraging social media to encourage healthy eating behaviors.

## Paper Context

- **Main findings:** The intricate and multifaceted drivers of food acquisition and consumption of Lao adolescents shed light on their tendency and preference to consume ultra-processed foods over fruits and vegetables.
- **Added knowledge:** The study reveals the diverse food environment in suburban Lao PDR, offering accessibility to both healthy and unhealthy foods. School food environments could inadvertently promote the consumption of ultra-processed foods.
- **Global health impact for policy and action:** A comprehensive, integrated public health nutrition strategy that targets school food environments, fosters caregiver involvement, harnesses positive peer influence through social media and acknowledges adolescents’ autonomy is needed to promote and cultivate healthy dietary habits among adolescents in Lao PDR and other similar low- and middle-income countries.

## Background

Adolescence is marked by rapid physical, mental, and cognitive changes, making optimal nutrition particularly important in this critical period of life [1]. Developing healthy dietary behaviors during these formative years is foundational for healthy food choices throughout the life course [2]. However, in low- and middle-income countries (LMICs), the transition in dietary patterns away from the consumption of traditional, unprocessed foods and minimally processed foods towards the increasing consumption of ultra-processed foods (UPF) is exacerbating the rising double burden of malnutrition, including among adolescents [3,4]. A recent analysis of the evidence on the double burden of malnutrition among adolescents from 57 LMICs found the prevalence of underweight to be 5.5%, whilst the prevalence of overweight and obesity was 21.4% [5].

Research synthesizing adolescent dietary behaviors in LMICs has found that adolescents consume low amounts of fruits and vegetables (FV) and high amounts of low-nutrient, high-calorie UPF [6,7]. High intakes of UPF are associated with an increased risk of non-communicable diseases such as diabetes, cardiovascular diseases, and obesity during adolescence and later in life [4,8]. Therefore, understanding the drivers of adolescent food acquisition and consumption practices, particularly related to the consumption of FV and UPF will be crucial to avert a future public health crisis in these settings, including among current and future generations [9].

Food environments are changing rapidly in LMICs as globalization, urbanization, and the growth of the food industry have collectively made UPF more readily available and accessible to adolescents, creating the conditions for the double burden of malnutrition [4,10]. Food environments are defined as ‘the interface within the wider food system, where people interact with food sources to acquire and consume foods’ [11]. For adolescents, the external food environment – such as school settings, retail stores, and informal vendors – and the household food environment provide the landscape within which inter- and intra-personal dynamics play out to shape food behaviors [12]. Although there has been a growing interest in improving adolescent nutrition by modifying their food environments to promote healthier diets [13,14], studies investigating the drivers of food acquisition and consumption practices among adolescents remain limited in LMICs [11,15]. This is the case in Lao People’s Democratic Republic (Lao PDR), where there is a paucity of evidence to date.

Lao PDR, like many other LMICs, is experiencing both an increase in the double burden of malnutrition among adolescents [16–18] and an upward trajectory in NCDs, substantially impacting morbidity and mortality within the population [19]. A cross-sectional study among adolescents in Lao urban areas reported prevalences of 10.3% underweight and 20.3% overweight [16], while a study conducted in Lao suburban areas reported prevalences of 3.7% thinness and 14.9% overweight and obesity, respectively [17]. Previous studies of adolescent diets in Lao PDR reported low levels of FV consumption, coupled with the high levels of UPF consumption, especially for sugar-sweetened beverages (SSB) [17,20]. Therefore, improving understanding of the drivers of food acquisition and consumption among adolescents will be crucial for the effective development and implementation of evidence-based interventions and policies designed to improve diets, nutrition, and health in this setting, as well as in other similar LMIC contexts. This study aims to address this research gap by exploring the drivers of food acquisition and consumption practices among adolescents in the suburban food environment of Lao PDR.

## Methods

### Study design

We used a Qualitative – Geographical Information System methodology to explore adolescents’ lived experience of their food environment and the drivers of their food acquisition and consumption. Adolescents were tasked with documenting their food environment using participatory photography [21]. The geotagged photos were subsequently mapped by the research team, and the maps and photographs were introduced into follow-up interviews and focus group discussions (FGDs) with participants to elicit narratives on drivers of food acquisition practices [21,22].

### Setting

The study site was Phonhong District, one of the 11 districts in Vientiane Province located 60 km north of Vientiane Capital in Lao PDR. The district has 37 secondary schools and a population of 65,200 [17,23]. This district was purposively selected due to low levels of FV consumption found in this setting in our previous study of 405 randomly selected adolescents from eight schools, with levels as low as less than four times in three days for fruits (94.8%) and vegetables (88.1%) [17]. Additionally, UPF consumption was found to be common among these adolescents, with 98.8% reporting UPF consumption within the previous week, accounting for 20.9% of total energy intake (Supplementary pp3–5). In this current study, we purposively selected two of these schools from previous study, including one located in the center of the district and one further away, with the aim of capturing the range of food environments in this setting (Supplementary Figure S1).

### Sampling

Purposive sampling was used to recruit participants. A target sample size of approximately 30 adolescents was anticipated to provide saturation in line with previous studies featuring similar designs [22,24]. To recruit participants, schoolteachers invited students to join the study. Teachers were provided with instructions and a brief script describing the study’s purpose and methods, which they used to explain the study to their classes when asking for volunteers. From the list of volunteers (*n* = 65 students), students were randomly selected to ensure a balanced representation of both sexes and age groups (12–14 years and 15–18 years). In total, 30 students were recruited (15 from each school).

### Data collection

**Figure 1.**
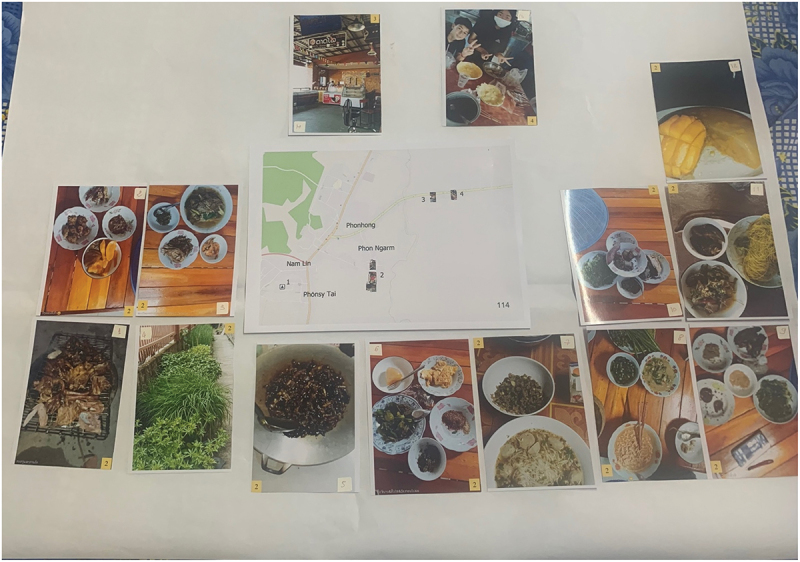
An example of a map and photos from a follow-up photo-elicitation interview (ID114, male, 16 years old). The map shows the locations where the photos were taken. Each photo is numbered with the number in the yellow box corresponding to the geotagged location on the map and the number in the white box indicating the order in which the photos were discussed.

The data were collected in two stages: 1) participatory photography and 2) follow-up photo-elicitation interviews and FGDs. In stage one, all participants were tasked with using smartphones (Brand: Xiaomi, Model: Redmi 9A Smartphone) to take photos of their food environment over seven consecutive days using SurveyCam, a GPS Camera application. We instructed participants to take geotagged photos of anything they considered important concerning purchasing, cooking, preparing, and eating of foods or beverages both inside and outside the home and where they sourced these foods. Participants then curated a sample of their photos that they felt represented their food environment and their food acquisition and consumption practices. In stage two, participants were invited to speak to the maps and curated photos, which had been prepared by the research team (Figure 1), and were invited to explain how they related to their lived experience of their food environment. Four additional follow-up FGDs (7 or 8 participants per focus group) were conducted to further explore the emerging narratives around common food sources and items captured in the photos, in line with established photo-elicitation questioning techniques [25]. FGDs were divided by sex into male and female groups. The follow-up photo-elicitation interviews and FGDs were conducted in the schools’ meeting rooms to ensure a private and uninterrupted setting. The duration of the photo-elicitation interviews ranged from 43 to 60 minutes, while the FGDs lasted between 60 and 80 minutes. Two researchers (TK and HX) collected the data in the Lao language between April and July 2022. Data saturation was determined when no new themes or points of discussion emerged from the analysis.

### Data analysis

Audio recordings from the photo-elicitation interviews and FGDs were transcribed verbatim in the Lao language, and transcriptions were subsequently translated into English. Transcriptions, photos, and maps were uploaded into NVivo for thematic analysis. We follow six phases of thematic analysis outlined by Nowell et al. (2017) [26]. Two authors (TK and MS) conducted the coding, and any discrepancies were resolved through the discussion. Deductive and inductive coding techniques were used in an iterative process. Deductive coding, informed by the conceptual frameworks published by Turner et al. (2018) and Contento and Koch (2020) [11,27], was used to generate initial set of codes and subthemes. Inductive coding was then used to identify additional in-vivo codes and subthemes emerging from the data. Preliminary codes and subthemes from these processes were compared, contrasted, and synthesized into the final set of themes grouped by external [11], intra-personal and interpersonal domains [27] in line with the multi-scalar nature of our findings. These themes were discussed and agreed by the first author (TK) and co-authors (MS, CT, and HX). The research team took a reflexive approach to the research process, documenting our assumptions and critically challenging our existing beliefs to identify and mitigate potential biases.

Participants’ geotagged photos were mapped using ArcMap software to visualize the locations where they were taken. All foods captured in participants’ photos were documented and assigned into one of three groups based on a simplified version of the NOVA classification system [28] to facilitate ease of mapping. Group 1 included photos containing UPF, Group 2 included photos containing non-UPF, and Group 3 included photos containing both UPF and non-UPF, as shown in Table 1.

**Table 1.** Food categorization of photos based on NOVA classification.

Food Group	NOVA Food Classification	Definition
1	Ultra-processed foods	Industrial formulations often include additives not commonly used in culinary preparation, resulting in hyper-palatability, increased profitability, and long shelf life. Examples include carbonated drinks, packaged snacks, ice cream, candies, cookies, cakes, sausages, and instant noodles.
2	Unprocessed or minimally processed foods	Natural foods that are minimally processed and free from added substances. Examples include fresh, squeezed, or dried fruits, vegetables, grains, eggs, milk, meat, and fish.
	Processed culinary ingredients	Substances derived directly from natural sources used to prepare, season, and cook. Examples include salt, sugar, butter, and vegetable oils.
	Processed foods	Food products made from a combination of natural ingredients and seasoning substances, processed using various preservation and cooking methods to enhance the durability of the natural foods. Examples include canned vegetables, fruits, fish, and cheeses.
3	Mixed unprocessed, processed, and ultra-processed foods	These foods are dishes that combine unprocessed, processed, and ultra-processed food. These dishes cannot be categorized solely as either processed or ultra-processed.

### Ethical considerations

The study protocol was approved by the Institutional Review Board of the School of Tropical Medicine and Global Health of Nagasaki University (Protocol No. 167) and the National Ethical Committee for Health Research of Lao PDR (Protocol No. 026/NECHR). Signed consent forms (for adolescents’ legal guardians) and assent forms (for adolescents) were obtained from all participants before data collection.

## Results

### Participant characteristic

Key characteristics of the participants are shown in Table 2. Approximately half of the participants were male and in the younger age group. The majority of participants were of Lao ethnicity. Half of the adolescents’ households owned rice paddies or fishponds, and more than 70% of them had gardens and livestock at home.

**Table 2.** Participant characteristics.

Variables	Sub-category	Frequency (%)
Sex	Male	17 (56.7)
	Female	13 (43.3)
Age	10–14	14 (46.7)
	15–18	16 (53.3)
Ethnicity	Lao	28 (93.3)
	Do not know	2 (6.7)
Rice paddy or fishpond ownership	Yes	15 (50.0)
	No	15 (50.0)
Home garden ownership (Fruits or vegetables)	Yes	22 (73.3)
	No	8 (26.7)
Home livestock ownership	Yes	27 (90.0)
	No	3 (10.0)

### Key food sources among adolescents and their households

**Figure 2.**
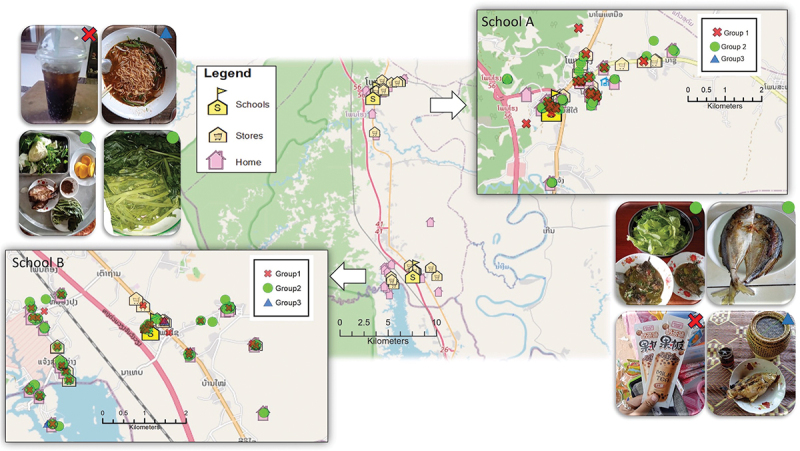
Reference maps displaying geolocation points from all food photos documented by participants. The map illustrates the type of foods consumed or available in their food environment at different locations, including their home, school, and stores. Group 1: ultra-processed foods; group 2: unprocessed, minimally processed, and processed food; group 3: photos contain both group 1 and group 2.

In total, participants captured 640 photos, of which 518 (81%) depicted food items or meals, 62 (10%) captured food activities such as cooking and eating with friends or family, and 60 (9%) captured food sources. Evidence from across the datasets revealed that adolescents acquire and consume foods from various sources, namely retail stores, supermarkets, school vendors, local markets, takeaway food stores, mobile food carts, gardens, ponds, gifts, and gathered from the wild. The most common food sources captured in the photos were retail stores/supermarkets (26 photos) and school vendors (13 photos). Figure 2 shows the foods documented at different locations over the seven days of the participatory photography activity, illustrating that Group 1 photos (i.e. UPF) were captured both inside and near schools.

### Drivers of food acquisition and consumption among adolescents

**Figure 3.**
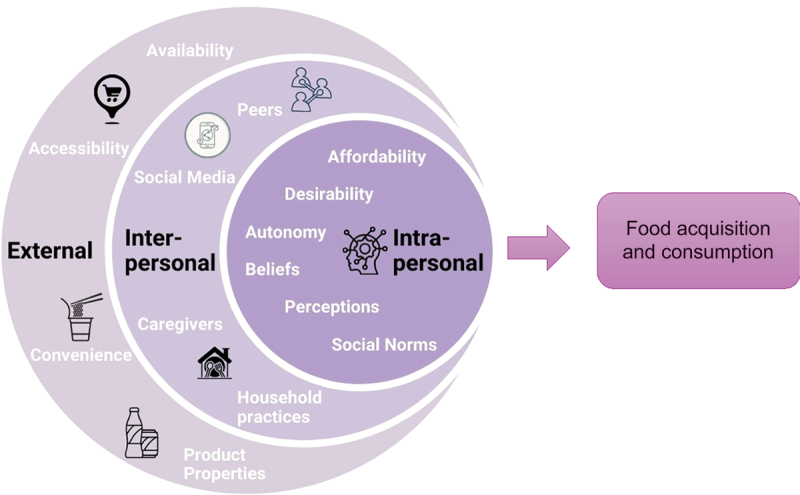
Diagrams of drivers for food acquisition and consumption of adolescents.

Drivers of food acquisition and consumption were complex and multi-scalar, involving interactions between external, interpersonal, and intrapersonal domains (Figure 3). The key themes resulting from the analysis were: 1) food availability and accessibility, 2) product properties and convenience, 3) peers and social media, 4) caregivers and household practices, 5) affordability, desirability, and autonomy, and 6) perceptions, beliefs, and social norms.

### Theme 1: food availability and accessibility

**Figure 4.**
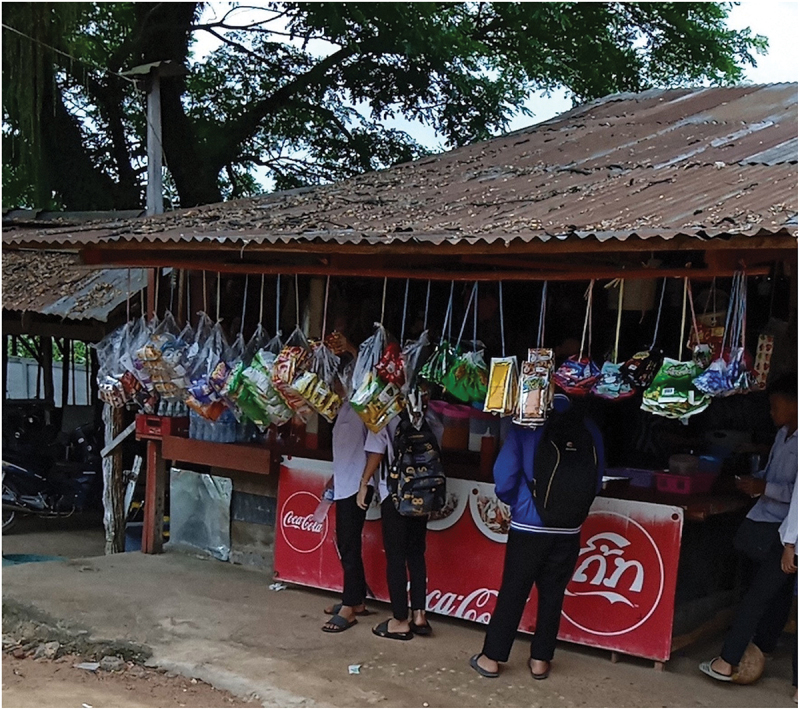
A photo of a school vendor taken by a study participant (ID106, male, 15 years old). The photo shows the snacks, beverages, and meals being sold. Banners promoting SSB are used to cover the customer facing side of the vendors’ tables.

Adolescents’ food acquisition and consumption practices were strongly determined by food availability and accessibility at their schools and homes. In particular, the frequent consumption of deep-fried foods, snacks, and SSB was attributed to their readily available and accessible nature at schools (Figure 4).

I came to school early in the morning but forgot to bring drinking water with me. Then I started drinking these drinks [SSB] from the school vendors and liked it. After that, I kept drinking them because I got used to it. (ID203, female, 12 years old, photo-elicitation interview)

If I go to school, I drink [SSB] every day. If I stay at home, some days I will have, some days I won’t…. Because at school, the shop is nearby, very near. (ID108, male, 16 years old, photo-elicitation interview)

In contrast, there were fewer options for FV. The most common were unripe sour fruits sold by school vendors and mobile food carts near the schools. Meal options were also described as limited at school vendors:

At school, apart from snacks and soft drinks, I think there is not a variety of food. There would be mainly rice top with some food, and rice noodle soup. That’s it. (ID109, male, 16years old, photo-elicitation interview)

Adolescents with motorbikes were able to travel further to acquire and consume lunch at other places, while those without any personal mode of transport were faced with little option but to consume lunch at school vendors or restaurants nearby the school.

There was a variety of food available at home. Adolescents reported consuming fresh ingredients, such as vegetables and fish, made available at home and sourced from local fresh markets and their home-grown products.

I cooked this because the eggs and veggies were already there in the kitchen; my mom bought it. (ID107, female, 14 years old, photo-elicitation interview)

This was our dinner. Almost all the ingredients such as vegetables were from our garden. We bought only mushrooms and Pak Kha [name of vegetable]. My grandmother got the bamboo shoot from the bamboo tree, and Yanang [name of vegetable] was from our garden. The papaya used for papaya salad was also from our garden. (ID108, male, 16 years old, photo-elicitation interview)

Seasonal fruits also featured:

I eat whatever fruits that are in season; for example, if it is mango season, then I eat many mangos. When it is coconut season, then I eat coconuts. (ID101, male, 13 years old, photo-elicitation interview)

### Theme 2: product properties and convenience

Adolescents typically preferred foods that were convenient to prepare, such as instant noodles, consuming them as main meals, as late-night snacks, or as an alternative option when they did not like the meals prepared by their caregivers:

I always stay late, so when I feel hungry, I would just get up and boil the instant noodle because, for me, it is the snack. It is simple to cook; just boil it. (ID103, female, 16 years old, photo-elicitation interview)

When my family cooks something I don’t like to eat, then I would eat instant noodles because it is easy to prepare. (ID107, female, 14 years old, photo-elicitation interview)

Packaged snacks such as potato chips, jelly, and cake were also considered convenient to eat and they were favored among adolescents:

I like snacks–any snacks, especially potato chips. I can eat it every day. I also eat Else [snack brand] sometimes when I am hungry. It is a kind of cake that is easy to eat and can make me feel full. (ID203, female, 12 years old, photo-elicitation interview)

**Figure 5.**
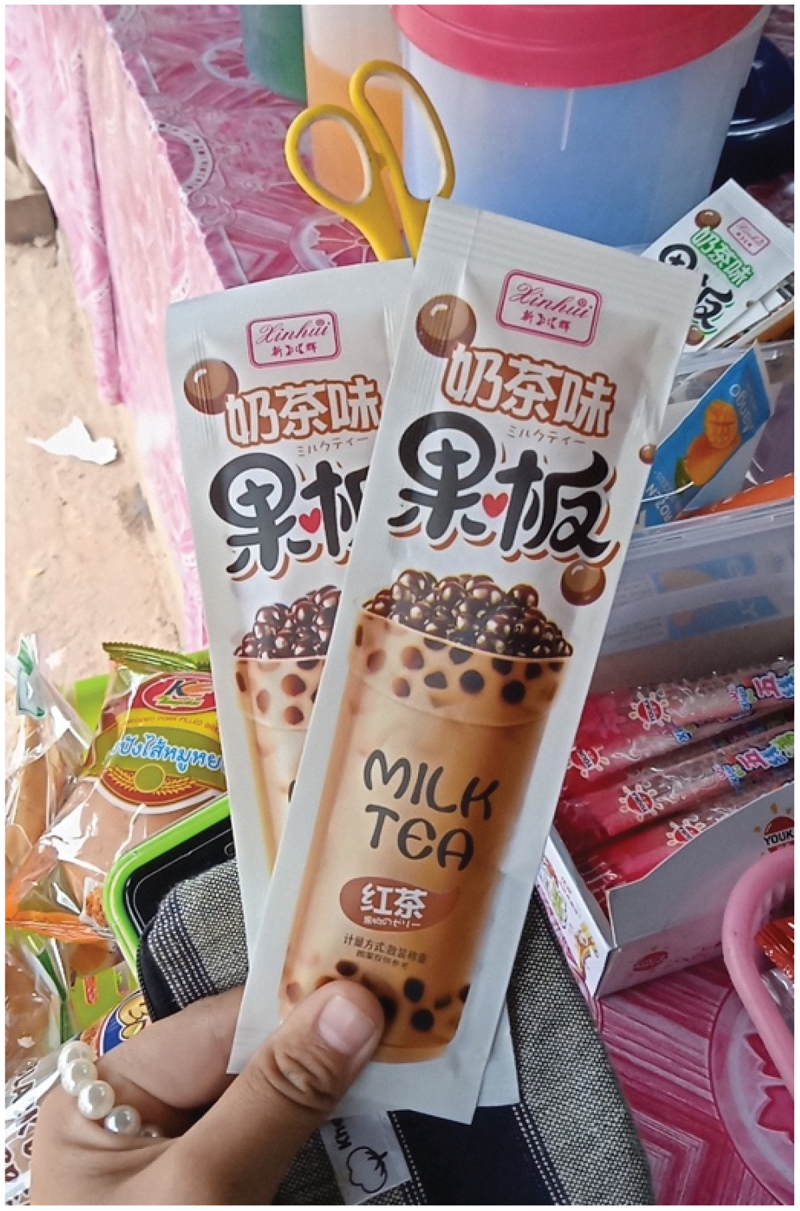
Photo of jelly snacks captured by a participant (ID111, female, 13 years old).

I like it [Jelly] because it is a kind of snack, like. it is easy to eat. (ID111, female, 13 years old, Figure 5, photo-elicitation interview)

### Theme 3: peers and social media

Peer influence was a strong driver of food consumption practices, as the adolescents spent most of their time during school hours with their friends, and those living in the same area could also spend time together in the evening or on weekends cooking food at home or dining at a nearby restaurant. Most adolescents (28 out of 30) had their own smartphones with accounts on multiple social networking services (SNS) such as Facebook, TikTok, and YouTube. Adolescents followed online food trends on these platforms:

We saw many people posted this food on Facebook and TikTok, so my friends and I just discussed with each other that we should cook it too. (ID102, female, 13 years old, photo-elicitation interview)

Adolescents described how their friends could also influence their decision to consume certain foods and even skip meals because they wanted to fit in with their peer groups:

When I see my friends eat something, I would want to eat it too. If all my friends eat that food, but I’m the only one who doesn’t eat it, it would be odd. (ID202, female, 13years old, photo-elicitation interview)

Most adolescents perceived that their friends were most likely to encourage them to eat snacks instead of FV:

I don’t think my friends would ever ask me to go eat fruits and vegetables. We usually ask each other to eat something like snacks, spicy food, and something trendy. (ID106, male, 14 years old, FGD)

However, one adolescent mentioned that friends could also be a role model for healthy eating habits and lifestyle:

But if we have friends with a healthy lifestyle, we can ask them for tips and advice. For example, I have a friend who likes exercising and eating healthy food. So, I always ask him about it. (ID105, male, 16 years old, FGD)

### Theme 4: caregivers and household practices

Caregivers and practices inside the households were found to be influential in a home setting. Caregivers usually cooked local dishes for meals that adolescents consume at home, which comprised vegetables, fish, meat, and various spices:

I like my parents’ dishes. My parents like to cook ‘Mok Pa’ [Steamed fish with herbs in banana leaves], and grilled meat […] Mok Pa would come with vegetables like steamed cabbage or boiled cabbage and mustard green stem vegetable. (ID111, female, 13 years old, photo-elicitation interview)

I eat my mom’s dishes every day. My mom doesn’t always cook the same old dish because we might get bored easily. She would keep changing the menu, but it would usually be the local dishes and fish menu. (ID102, female, 13 years old, photo-elicitation interview)

Adolescents described their caregivers’ influence on their food behaviors:

My mother always says, “If you don’t eat proper meals, you will be sick like you used to be. You will have a fever. You will have stomach problems. You will be unhealthy”. I don’t like my parents complaining, so I have to always have meals with my parents. (ID205, male, 13 years old, photo-elicitation interview)

Most households grew vegetables or fruits in their home gardens and raised livestock, including chickens, ducks, and fish. Some adolescents were engaged in home gardening and livestock care and used these home-produced ingredients when cooking:

It is bamboo shoot soup and papaya salad. I ate it at home. My mom made the bamboo shoot soup. I made the stir-fried vegetables and papaya salad. (ID113, female, 15 years old, photo-elicitation interview)

Engaging in preparing, cooking, and eating meals with other family members was also described as an enjoyable experienced by some adolescents:

I like this one the most [pointing at photo], “Kapao” [pork with basil]. I liked it because that day I cooked it together with my dad. It was delicious! My dad also likes cooking. I prepared the pork and washed vegetables for him, and he cooked and seasoned it. (ID111, female, 13 years old, photo-elicitation interview)

### Theme 5: affordability, desirability, and autonomy

Adolescents’ decision about food acquisition was influenced by its affordability. They often received pocket money from their caregivers, and the amount received affected their food purchases. While some received enough pocket money to buy snacks at school, others did not and had to rely mainly on foods available at home:

I have to restrain myself from eating food or snacks I like because they are sometimes too expensive. In these pictures, the foods we ate were mostly from our garden or what we could find around our house. Whatever we can find, we have to eat those. Like these grilled fish, we took the fishes from our pond. (ID105, male, 16 years old, photo-elicitation interview)

Adolescents who received enough pocket money could use that money to buy the snacks or the foods they like at the stores at schools, restaurants, and other market-based vendors:

I eat them [shrimp crackers] every other day depending on how much money my mother gives me. (ID104, male, 13 years old, photo-elicitation interview)

Although adolescents mostly relied on their caregivers for pocket money and their food consumption at home, they nevertheless demonstrated a degree of autonomy in buying, preparing, and cooking food for themselves and their households. A few adolescents mentioned that they tended to avoid what they considered to be foods for adults as they viewed them as ‘foods for old people’ and rather preferred ‘modern foods’ such as noodles, fried chicken, pizza, and others. When their households prepare foods that they do not like, some adolescents described buying or cooking an alternative option:

I cook food for my father and cook separate food for myself because I don’t like something like that. The taste is strange. I don’t like the ingredients [herbs and vegetables with a strong smell, or bitter taste]. I don’t like that kind of food for older people. I like to try new things. Mostly, I like to try modern food. (ID109, male, 16 years old, photo-elicitation interview)

Others described rejecting foods prepared by their caregivers:

I hate vegetables. When my family makes vegetable dishes for me, I just buy other foods to eat. (ID115, male, 16 years old, FGD)

Most adolescents chose foods based on their perceived desirability. Although they could not clearly express the reasons for desiring certain foods, some participants associated it with the taste and pleasure of consuming specific foods or drinks:

I like to drink this soft drink because it is sweet. I drank it in the evening after I finished playing football. (ID215, male, 15 years old, FGD)

Many of them were conscious of healthy food practices but found adherence challenging due to the desirability of unhealthy food:

When I tried to eat healthier, it lasted for a short period. I could do it on the first day. On the second day, I would be craving for the food I like–unhealthy one I mean, and that’s when I failed to change my food habits. (ID109, male, 16 years old, FGD)

Some adolescents explained how they were able to influence their caregiver’s food acquisition practice to purchase foods that they desired:

My mom usually buys it [instant noodles] to stock at home because my brother and I like to eat it. (ID103, female, 16 years old, photo-elicitation interview)

I like deep-fried food, for example, deep-fried chicken drumstick with flour battered, so my mom bought it for me sometimes from Vientiane Capital. (ID109, male, 16 years old, photo-elicitation interview)

### Theme 6: perceptions, beliefs, and social norms

Perceptions and beliefs related to foods also influenced their acquisition and consumption. Some adolescents described how they avoided foods they considered harmful to their health and body while consuming foods they thought were beneficial to them:

I always eat breakfast because it prevents me from feeling hungry when I get to school in the morning. It nourishes our brain because we must use our brain a lot when studying. (ID103, female, 16 years old, FGD)

I ate dragon fruit. On that day, there were also apples, pears, and mangoes. I like all of them. I think they are important because they help with digestion and defecation. (ID110, male, 13 years old, photo-elicitation interview)

I avoided soft drinks because if I drink too much, it might weaken my bones and cause my bones to break easily. (ID212, male, 15 years old, FGD)

Avoidance of unhealthy food was explicitly expressed by adolescents who were concerned about their body image, particularly when they experienced teasing and criticism of their body weight:

I don’t eat them often because I fear gaining weight since they are chips. They are delicious, though. This one also [pointing to the soft drink] I am afraid of gaining weight, so I don’t drink much […] Because people always say why I am getting fatter. I was small and thin before. My friends always ask me why I am fat now. (ID111, female, 13 years old, photo-elicitation interview)

I wanted to control my weight, so I tried intermittent fasting. My friends, parents, and others always tell me to lose weight. Sometimes they tease me, and I don’t like it. The most weight I had was 108 kg. I have not measured it recently; it might be 100 kg now.’ (ID101, male, 17 years old, photo-elicitation interview).

## Discussion

Our study highlights the multi-scalar and complex drivers of adolescent food acquisition and consumption in suburban food environments of Phonhong District, Lao PDR. The key themes that arose from this study were 1) food availability and accessibility, 2) product properties and convenience, 3) peers and social media, 4) caregivers and household practice, 5) affordability, desirability, and autonomy, and 6) perceptions, beliefs, and social norms. Addressing these multifaceted drivers is essential for the promotion of healthier dietary habits among adolescents in this context.

Our findings related to the importance of food availability and accessibility, as well as product properties and convenience are consistent with previous studies that the external and personal food environment domains influence food practices in LMICs [14,29,30]. The widespread availability of UPF within schools, where adolescents predominantly spend most of their weekdays, is a strong influence on their consumption of these products. While schools should be promoting healthy food practices, results from this study support findings from prior studies in LMICs that consistently demonstrate the widespread availability and consumption of UPF in school environments [11,24,31]. The high availability, low cost, and extensive promotion of UPF by wholesalers may influence school vendors to prioritize these products for higher profits [32]. Public and school-level policies are needed to avert high levels of consumption of such products [33,34]. Combining restrictions on the availability of unhealthy products, providing healthy school meals, and subsidizing fruit and vegetables has proven effective in promoting healthy dietary habits among adolescents in high-income countries [35]. However, implementation challenges may arise when adopting these strategies in resource-limited LMIC settings [35]. Price control (i.e. increased taxation) of UPF may reduce the affordability of these products in the school food environment in LMICs. Evidence from both HICs and LMICs suggests that a 10–15% tax increase on such products can result in reduced consumption levels [36,37]. However, the successful implementation of these taxation schemes in LMICs requires well-designed taxes, political support, and public acceptance [38].

Our finding on the important role that caregivers and household practices play in shaping adolescents’ food acquisition and consumption practices is also consistent with the wider literature [12,14]. Caregivers play an essential role in influencing adolescents’ food behaviors, both through the food and guidance that they provide. Therefore, the attention and awareness of caregivers/parents regarding their children’s eating habits are crucial to prevent the intake of unhealthy food and to promote a healthy home food environment [39]. The household’s food culture and the proactive efforts of parents in offering advice on nutrition and health, along with providing healthy food options, could positively influence adolescents’ FV consumption. Maintaining the existing practices of home FV cultivation and preparing home-cooked meals also supports adolescents’ FV intake [40,41].

Our study found peer influence to be a crucial interpersonal driver of food practices among adolescents in Lao PDR, including through social media. This finding is consistent with previous qualitative studies that have revealed how adolescents, especially in mixed food environments (featuring both traditional and modern food sources), tend to prioritize the social value of food over its function as a basic necessity [42]. Adolescents seek peer approval and social identity, which makes peer pressure not only an important factor in underage alcohol drinking behavior in Lao PDR [43], but also a significant driver of their food behaviors [44–46].

The digital food environment is emerging as a significant typology within food environment research, reflecting the influence of digital media and online platforms on food acquisition and consumption behaviors [47]. Food marketing in the media generally promotes positive attitudes toward unhealthy foods [48]. However, social media interventions have been shown to increase FV intake while reducing unhealthy snack consumption among adolescents [49]. Given the significant sway of the digital realm and peer influence among adolescents, peer-enhanced social media interventions that employ influencer strategies could potentially be utilized to promote healthier eating habits [50]. Much of the evidence on the role of digital food environments to date is from research in HIC contexts [47], and as such there is a need to explore the impact of digital food environments on adolescent food acquisition and consumption behavior in LMICs, including the ways in which interventions may leverage digital tools to improve public health outcomes.

Our results related to the individual or intra-personal level of drivers of food behaviors are consistent with existing literature on personal food environment and individual determinants of food behaviors [14,30,42,51]. Adolescents are assertive in taking control of their behaviors based on their perspectives and beliefs, motivating them to seek autonomy and social belonging [42]. Recognizing their autonomy is therefore important when designing health and nutrition promotion programs. For example, school-based activities, family practices, peers, and social media influences aimed at developing autonomy and skills in cooking and preparing healthy food from their home-grown FV produce. On the other hand, adolescents shared negative concerns about weight gain and body image [52]. Although fear of weight gain might make them avoid unhealthy foods, their lack of understanding and misconceptions about weight loss could potentially lead to harmful weight loss practices and other psychological effects [53,54]. These findings suggest that in addition to a supportive food environment, it is imperative to improve the knowledge and awareness of healthy dietary practices among adolescents to enable them to make informed decisions in relation to their diets, nutrition, and health.

This research contributes new insights by emphasizing the role of school food environments, peer networks (both physical and digital), the persistent influence of family practices, and adolescents’ autonomy in acquiring and consuming food. In many LMICs, there is a notable gap in holistic interventions that comprehensively address the diverse factors influencing adolescents’ food environments. Current strategies often focus on isolated aspects, such as school-based nutrition programs or general health education [35,55], without integrating the full range of external, interpersonal, and intrapersonal drivers of food acquisition practices. In Lao PDR, there has been a tendency for isolated interventions focusing on aspects such as school feeding programs or general nutrition education for school-age children. Although the government recognizes the importance of adolescent nutrition, the current national nutrition policies do not specify the need for a holistic, integrated intervention to improve the healthiness of food environments for adolescents [56]. This fragmented strategy overlooks the interplay between school food environments, peer and family influences, and the impact of social media. Our findings call for multifaceted, context-specific interventions, particularly in school food environment policies and digital health promotion. A system approach that combines complementary multi-scalar interventions to target improvement in adolescent diets, nutrition and health should be pursued in Lao PDR, as has been called for in other LMICs [33].

### Strengths and limitations

A key strength of this study is the participatory methodology, which enabled an in-depth exploration of the drivers behind food acquisition and consumption among adolescents. The use of participatory photography allows participants to visually express their experiences and perceptions, while follow-up photo-elicitation interviews and FGDs, incorporating geolocated maps and photos, provide opportunities for detailed reflection and elaboration on their food practices. This combination of data collection techniques enriches the analysis, offering nuanced insights into Lao adolescents’ food environments and dietary behaviors. The triangulation of data from different methods adds to the credibility of the study [26]. Furthermore, prolonged engagement with participants and the inclusion of member-checking during follow-up interviews ensured that the results accurately reflected the adolescents’ perspectives. Our study also contributes to a small number of pioneering studies that have used participatory photography for food environment research in an LMIC contexts [21]. It demonstrates a successful application of participatory photography with adolescents in suburban Lao PDR, evidencing its broader potential beyond high-income country contexts and underlining its adaptability and efficacy across diverse socio-economic landscapes.

Throughout the research process, we made efforts to ensure the trustworthiness of our qualitative study [26]. However, we acknowledge certain limitations, such as the context specificity of our in-depth qualitative study in Lao PDR, which may limit the transferability of the findings to other settings. To account for this limitation, we provided detailed descriptions of the study process and context, enabling other researchers to assess the relevance of the findings to similar settings, particularly in LMICs. We also acknowledge the influence of our own perspectives on the findings. To acknowledge this, we kept a detailed record of our thoughts, values, and assumptions and engaged in continuous reflexivity throughout the research process, ensuring that we critically examined how our viewpoints might affect interpretation and analysis.

## Conclusion

The findings of this study align with broader findings from food environment research focusing on adolescents in LMICs, while also contributing several unique insights specific to the Lao PDR context. We would like to highlight the complex and multi-scalar nature of the interplay between external, interpersonal, and intrapersonal food environment domains. Specifically, the intake of ultra-processed foods was determined by their accessibility in schools, peer pressure, social media promotion, affordability, and desirability. In contrast, the intake of FV was largely influenced by parental food practices at home and the adolescents’ perceptions of the healthiness of FV. To enhance adolescent diets, nutrition, and health, a system approach that bundles complementary multi-scalar food environment interventions should be pursued by the government and supported by non-governmental organizations. These interventions include restricting the availability of UPF in school food environments, promoting of home gardening, encouraging caregiver involvement in adolescent food habits, and leveraging positive peer influence through social media to foster food and nutrition awareness, enhance knowledge, and cultivate healthy dietary habits among adolescents.

## Data Availability

The datasets used in this study are available upon request to the corresponding author.
